# What are patients’ expectations of orthodontic treatment: a systematic review

**DOI:** 10.1186/s12903-016-0182-3

**Published:** 2016-02-17

**Authors:** Jie Yao, Dan-Dan Li, Yan-Qi Yang, Colman Patrick Joseph McGrath, Nikos Mattheos

**Affiliations:** Department of Oral Rehabilitation, Faculty of Dentistry, The University of Hong Kong, Prince Philip Dental Hospital 4/F, 34 Hospital Road, Sai Ying Pun, Hong Kong, SAR China; Department of Orthodontics, School of Dentistry, Nanjing Medical University, HanZhong Road 140, NanJing, 210029 China; Department of Orthodontics, Faculty of Dentistry, The University of Hong Kong, Prince Philip Dental Hospital 2/F, 34 Hospital Road, Sai Ying Pun, Hong Kong, SAR China; Department of Periodontology and Public Health, Faculty of Dentistry, The University of Hong Kong, Prince Philip Dental Hospital 3/F, 34 Hospital Road, Sai Ying Pun Hong Kong, SAR China

**Keywords:** Patients’ expectations, Orthodontic treatment, Malocclusion, Systematic review

## Abstract

**Background:**

What patients expect to happen during treatment or benefit from the treatment might influence the subsequent factors such as treatment outcome, patient satisfaction, patient’s cooperation as well as compliance. The aim of this systematic review is to assess the role of patients’ expectations from orthodontic treatment.

**Methods:**

A systematic literature search of four databases Pubmed, Cochrane, Web of Science and PsychINFO was conducted following *PRISMA* guidelines. Studies reporting expectations regarding orthodontic treatment were selected and a narrative review was conducted. The quality of study was rated according to *STROBE* statements and the methodology as well as key findings were summarized.

**Results:**

Thirteen studies (14 papers) were finally included for analysis. Among them, only one was a randomized control trial, while the rest included one cohort study, two questionnaire-developments and ten cross-sectional studies. The *STROBE* quality of reporting scores of the studies ranged from 12 to 18. Seven papers described expectations of the treatment experiences, along with seven talking about benefit expectations from the treatment. Dental appearance and function improvement were most expected in studies relate to the treatment benefits.

**Conclusions:**

Orthodontics appears to have adopted various standardized questionnaires. However, most of them are poor in the quality of methodology and results analyses, which prohibit synthesizing sufficient evidence to help identify which factors influence patient expectations. The evidence of “expectations” affecting treatment outcomes is not found in current research. Future studies are needed to better understand the impact of “expectation” on the treatment both theoretically and experimentally.

**Electronic supplementary material:**

The online version of this article (doi:10.1186/s12903-016-0182-3) contains supplementary material, which is available to authorized users.

## Background

With increased focus on the delivery of “patient-centered” care, current research has been increasingly set on investigating the impacts of psychosocial factors in clinical treatment outcomes. As often reported, “What patients think will happen can influence what does happen over the clinical course” [1]. Expectation, as one of the important psychological factors, is often found to influence patient’s evaluation of the quality of treatment or final satisfaction with the treatment outcome [2]. This is even more relevant within long-term treatments where aesthetics is a significant component of the treatment outcome. One hypothesis is that the patients perceive treatment effectiveness through comparing their expectations with the actual experiences [3]. The gap between expectations and reality possibly can influence cooperation in treatment regimens [4]. Furthermore, unfulfilled expectations could contribute to dissatisfaction, which is more likely to lead to poor compliance as well [3, 5]. Seen from a positive perspective, expectation is also regarded as a catalyst for improving the success of treatment. For instance, there is evidence of expectations generating positive outcomes in patients who accept placebo interventions [3, 6].

Orthodontic treatment is a type of care which often involves young adults or minors, requires higher compliance for long term than other treatment, and the patient are often involved in decision making (parents, custodians). Previous studies have revealed that patient/parent expectations from orthodontic treatments are to get better facial or dental appearance, dental health and oral function [7, 8]. Improvement of “social competitiveness”, attractiveness and psychological confidence are also perceived benefits from orthodontic treatment [9–11]. However, there is another kind of expectations related to the treatment process and experience in itself [12]. For example, if patients/parents do not have a clear understanding of the duration of treatment, possibility of removing teeth, possible pain and discomfort with eating, speaking and cleaning teeth, reaching satisfactory treatment outcomes is more challenging than with people who have had sufficient mental preparation prior to treatment [13–15].

It appears that studies regarding with quality assessment of health care have taken for granted that fulfilled expectations will guarantee patients’ satisfaction. However, the current evidence for patients’ expectation is still highly questionable, which needs much more theoretical and empirical investigation. Until now, we still do not know whether awareness of patient expectations prior to treatment and correction of unrealistic perceptions could enhance the quality of health care and improve satisfaction with the final treatment outcomes. In order to answer these questions, a systematic review of the literature was conducted to critically examine current research of the role of patients’ expectations within orthodontic treatment. Three focus questions were investigated: {1} What are patient expectations related to orthodontic treatment? {2} Which are the main factors that determine of “patient expectations” from orthodontic treatment? {3} Can patient expectations affect the process and outcomes of orthodontic treatments and in what ways?

## Method

### Study protocol and eligibility criteria

A systematic search of the literature was conducted in order to identify research within the role of patients’ expectations from orthodontic treatments. Two independent researchers conducted the search according to the *PRISMA* guideline [16]. Studies were initially included if they met the following criteria:

1. Human subjects were investigated with regards to their expectations from orthodontic treatment.
2. Experimental studies (randomized or not, prospective, retrospective and cross sectional) with qualitative and/or quantitative analysis. Measurement can be conducted by surveys with a general question or a series of questions focusing on specific clinical situations. The instrument can be unipolar or bipolar scale. For example, continuous scale such as visual analogue scale (VAS) or five-point Likert scale.
3. The description of patients’ expectation fulfilled the definition of ***predicted expectation****s* in Thompson and Sunor’s article [17]. That is a kind of realistic and practical belief that something will happen actually during process or after the treatment completed. Usually, these expectations result from personal experiences, information from other people or social media.

### Search strategy and data resources

Since patient “expectations” represent a rather new area in dental research, no suitable MeSH term was available. A search was broadly employed to identify as many relevant studies as possible. The overall search strategy was defined used the text words”expectation” or “anticipation”, combined with “orthodontic treatment” and MeSH terms “malocclusion”. The search syntax was displayed [Additional file 1].

Literature search results originated from the online databases: Pubmed, Cochrane, Web of Science and PsychINFO. The period of publishing was set from 1995–2015, and the final search was run on August 2015. Any type of literature with the patients’ expectation topic in English was included to the initially screened and the hand search extended to the references listed in the included studies.

### Study screening and data extraction

Two reviewers screened the title and abstract of each citation independently to determine whether the study would be further retrieved in full text. Based on the pre-determined eligibility criteria, studies with a clear description of the aim, method (e.g. sample type and size, study design) and result were considered. Full-text copies of the possible eligible studies were retrieved. After the assessment of the full text, decision was made by the two reviewers for final selection. The inter-reviewer agreement for each eligibility citation was calculated. Disagreements were resolved by discussion in a series of stages. In case of disagreement, other co-authors were involved in discussion until consensus was reached.

Once the studies were selected for final analysis, the following data of each study was extracted by one reviewer: author, year of publication, name of journal, subjects (age, diagnosis, and previous orthodontic experiences), study design, measurements (instrument, questionnaire items and main factors) and results. The second reviewer controlled the extracted data and if any objection or disagreement occurred, this was resolved by consensus. Meta-analysis of the results was not possible due to the wide range of study designs. Thus a narrative synthesis was undertaken.

### Analysis and quality assessment

The criteria in *Strengthening the Reporting of Observational Studies in Epidemiology (STROBE)* were utilized to evaluate the study quality [18]. The *STROBE* statements represent the quality standards of observational studies (cohort, case–control and cross-sectional studies). The 22 items in *STROBE* provided guidance to assess the title, abstract, introduction, methods, results and discussion sections. Two investigators rated the score for each study (fully met = 1; Partial met = 0.5; N/A or Not at all = 0). The mean scores of two raters were recorded as the final quality score.

## Results

### Study selection

**Fig. 1:**
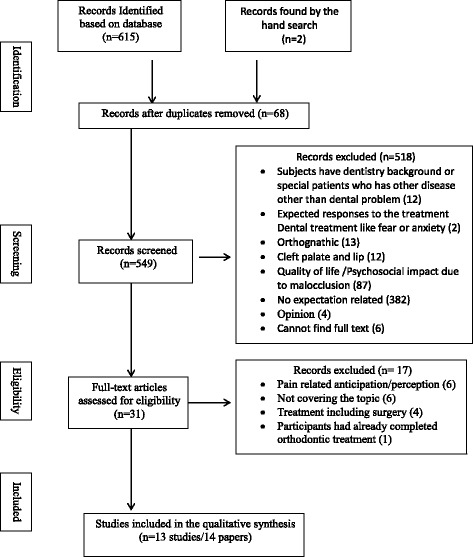
Phases in the development of eligible literatures

The selection process based on the *PRISMA guideline* is presented in Fig. 1. Four databases initially provided a total of 615 citations. Two studies were chosen from references by hand search. After adjusting for the duplicates (68 studies), 549 studies were further remained. The first round screening discarded 518 studies through evaluating the titles and abstracts (inter-reviewer agreement, kappa = 0.78). Studies were excluded because of:

1. Not covering the topic at all in the study aims (382 studies)
2. Investigating only quality of life/psychosocial impact due to malocclusion (87 studies)
3. The study sample having dental background or special diseases other than involving dental problems (12 studies)
4. Investigation in clefts of the lip or palate, craniofacial syndromes, and orthognathic Problems (25 studies)
5. Investigating only response expectations to the treatment like fear or anxiety (2 studies). Response expectations are investigated in systematic desensitization therapy and they are anticipations of automatic reactions to particular situational cues [19].
6. No full text available (6 studies)
7. Only opinions (4 studies)

The full texts of remaining 31 studies were examined in detail. 17 studies were excluded during the final round screening because of various reasons (inter-reviewer agreement, kappa = 0.91) [Fig. 1]. Studies without showing the descriptions about orthodontic related treatment outcomes or process were excluded. In addition, studies with participants that have already completed treatment and including oral surgeries were excluded as well. Two studies were chosen from references by hand search [20, 21]. Therefore, 13 studies (14 papers) published from 1997 to 2015 were finally identified. The eligibility criteria were consistent during all the stages of screening.

### Study characteristics

The search results revealed that a wide variety of study designs had been used to examine the patient expectations of orthodontic treatment. Among the 13 studies (14 papers), only one was randomized control trial with intervention, the other were observational studies including one cohort study, two questionnaire-developments, and ten cross-sectional studies. All of them were quantitative studies using questionnaires to measure interest parameters. Additional file 2: Table S1 displays the summary of the study characteristics and main findings of each study. The diverse types of questionnaires and primary parameters are listed in Additional file 3: Table S2. Due to the discrepancy of the focus among studies, the analysis was organized into two parts:

- *Expectations with regards to the treatment process experience and impact on quality of life*
- E*xpectations with regards to the treatment outcome and benefits*.

All twelve observational studies were rated with the *STROBE* score ranging from 12 to 18 (total score = 22) [Additional file 2]. Based on the content in *STROBE*, the highest score (≥9) was given for the title, abstract, introduction (background and objectives), outcome data and discussion (key results). The lowest score (≤2.5) was given for bias in description, study size explanation, and discussion (interpretation, generalizability). For example, the abstract of Sadek et al.’s study did not present the results in numerical form, which offended the criteria of *STROBE* [22]. The score of 0.5 would thus be recorded.

- *Expectations with regards to the treatment process experience and impact on quality of life*
  There were seven studies (8 papers) primarily investigating patient expectations regarding the orthodontic treatment experiences [12, 22–27] or impact on the related quality of life [20]. Five papers applied the same questionnaire, which was developed by Sayer and coworkers in 2006 [12, 22, 25–27]. This questionnaire was present with acceptable validity and reliability. Two studies were conducted as a survey with a limited sample size of 50 and 60 subjects [23, 24], respectively. Finally, Zhang et al. adopted the Child Perception Questionnaire (CPQ_11-14_) to measure patient expectations on oral related quality of life during treatment procedures [20].
  Nasr et al. divided patients into two groups before they consulted with orthodontists with the treatment [27]. The case group was assigned the leaflets with information of orthodontic treatment, from where patients were anticipated to get basic knowledge related to the treatment procedures and benefits. In control group, patients got leaflets with no reference to orthodontic treatment procedures. The expectations of patients were measured again after the consultation procedures. However, the results found no significant differences between intervention and control groups. Another finding in this paper was that boys and girls had similar expectations of orthodontic treatment, which was consistent with Hismstra et al.’s report [26]. Expectations of parents and their children were commonly compared among studies [22, 25, 26]. Sayer et al. found that expectations of parents were more realistic than the children [25]. For example, parents were better aware of dietary and drinking restrictions during wearing the orthodontic appliances. The conclusion was then confirmed by Hismstra et al. and Sadek et al., albeit the results in Hismstra’s study showed more aspects of differences [22, 26]. Additionally, Hismstra et al. compared samples from Dutch and UK with the same questionnaire and concluded that different health systems would influence patient expectations [26]. Sayer et al. scrutinized ethnic diversity and stated that the culture difference was one factor resulting in variances [25]. This was further found in Saderk et al.’s findings, where black British and white British patients showed different expectations of the initial appointment [22]. Black British patients expected to have a brace fitted instead of just a consultation discussion.
  Zhang and the coworkers utilized the 37 items Child Perception Questionnaire (CPQ_11-14_) developed by Jokovic et al. in 2002 [20, 28]. This questionnaire was for pediatric oral health related quality of life (OHRQoL) in age of 11–14 years old, which had been assessed for validity and reliability. Four factors were derived from responses: *oral symptom (OS); functional limitation (FL); emotional well being (EWB)* and *social well-being (SWB)*. 197 children with the mean age 13.1 years old were measured pretreatment, then they were followed at 1 week, 1 month, and 6 months after insertion of the fixed appliances. The results indicated the impact on OHRQoL after insertion of fixed orthodontic appliances was considerately less than what child patients expected. However, referring to the comparison of data from different time points, the authors found OS and FL were significantly less compromised than anticipated. EWB and SWB did not compromise in reality as expected at all-time points of treatment.
  Eight papers with four kinds of questionnaires measured patient expectations on orthodontic treatment experiences [Additional file 3]. The primary factors investigated in all these questionnaires were anticipated changes in social activity [12, 22–27]; the duration of treatment [12, 22–27]; types of treatment [12, 22–27]; pain problems [12, 20, 22, 24–27]; situation during the initial appointment [12, 22, 23, 25–27]; frequency of revisit [12, 22, 23, 25–27]; restriction in oral function such as eating [12, 20, 22, 24–27]; restriction in oral hygiene [23, 24]; relationship with orthodontists [23, 24]; proficiency of dentist [24] and treatment complications [23].
- E*xpectations with regards to the treatment outcome and benefits*.
  Six studies were grouped in this subtopic. The questionnaire of Bos et al., Tung et al. and Wezel et al.’ s study were selected from Kiyak et al.’s studies, which were developed for measuring what patient expected to benefit from orthognatic surgery [7, 29–32]. Four factors were identified through principle components analysis: *general well-being, self-image/appearance, future dental health* as well as *oral function*. In Bos et al. and Wezel et al.’s research, the impact on patient satisfaction with general facial/dental appearance, and the effect of demographic charters on patient expectations were investigated through multiple regression analysis [7, 30]. As reported, the dental related satisfactions of patients significantly influenced their expectations on general well-being, improvement of self-image/ appearance as well as future dental health. Age was significantly related to patient expectations on self-image, yet this significance was not affected by the gender. Tung et al. compared variables of parents and patients’ expectations from treatment [29]. The authors claimed that although both parents and patients had high expectations on improvement of self-image and oral functions but little on social life and general health, the parents seemed to expect greater improvement than their children.
  Petrone et al. modified the questionnaire developed by Bennett and the coworkers in 1997 [8, 33]. The questionnaire in Patrone’s study was composed of ten items originating form Bennett et al.’s 52-items scale. Bennett et al. [8] constructed two versions of expectations questionnaire, one for parents and the other for the orthodontists. Through the qualitative interview, pilot test and factor analysis, four factors were yielded namely: *benefits of treatment; long-term risks; short-term risks* and *inconvenience*. The four main factors could explain 45 % of whole variances. As for parents, expectations of benefits from treatment were highest and they were found related to the family income, father’s education level, and the gender of respondents. Petrone et al. tested expectations of 92 patients aged more than 18 years old 3 months before the treatment [33]. The patients included in this study should pay full treatment fees all by themselves. The associations between cost, malocclusion severity and benefits expectations were analyzed. Two main expectations were summarized as *straightness of the teeth* and *general appearance improvement*. The results demonstrated that patient expectations of benefits from treatment were significant associated with the severity of malocclusion but not the treatment fees. Finally, Tuncer et al. only adopted one question with three choices to measure patients’ expectations and they found dental aesthetics was the determinant for orthodontic treatment outcomes for more than 50 % of patients and parents [21]. Especially, parents with higher education level would pay more emphasizes on oral function.

## Discussion

In general, dental appearance and function improvement were the uppermost expectations regarding orthodontic treatment outcomes [7, 8, 12, 20–27, 29, 30, 33]. The subjective self-concept, age and malocclusion severity of the patients were main reasons for seeking treatment, which impacted on the expectations as well [7–9, 30, 33]. From the current research, both parents and children patients appear to have basic knowledge and practical expectations of the orthodontic treatment. However, parents seemed to be more realistic than children with regards to the impact of treatment procedures [21, 22, 25, 29]. This might be on the grounds that orthodontic treatment is relatively pervasive in nowadays and parents usually have more information resources from friends, relatives, Internet etc. [22]. The hypothesis that females have higher expectations than males was not confirmed in current evidence [7, 25–27, 30]. However, background such as ethnicity, education level and different social health systems were singled out as potential factors influencing patients’ expectations [7, 21, 22, 25, 26].

Almost all subjects included in studies are newcomers of orthodontic treatment. Some of them are visiting the orthodontist for the first time or referred from general practitioners. Thus, the majority of their expectations are based on the existing knowledge or information irrespective of the accuracy of the source. Especially the adolescents, who are probably more easily influenced by the notions of “beauty” advocated by social media, could overestimate the treatment outcomes and underestimate the complexity of procedures [34]. For example, the periodontal or TMJ complications, pain perceptions during wearing appliances are sometimes beyond their expectations. The commitment of long-term maintenance, requirements of keeping oral hygiene are usually very demanding on patients’ time [35]. Thus, it is essential to communicate in detail about the risks and benefits before starting the treatment. Noted that, there is a considerable proportion of adult patients who are also surprised, when they are told that the orthodontic correction will not be long lasting without retention [36]. Moreover, a study found that more than 30 % of the patients would consider, or had already undergone other cosmetic dental or surgical procedures such as tooth whitening, breast enlargement, etc. [37]. This result suggests a category of patients who are less satisfied with other aspects of physical appearance. Recognizing such a group of patients at an early stage of treatment is critical, because these patients might have unreasonable expectations, and the dentists will have to clearly state what can, and cannot, be achieved with orthodontics. However, none of the studies included in this systematic review addressed the risks and management of unrealistic expectations. Another important point highlighted in Zhang’s cohort study, is the gradual decrease of patient expectations during different treatment stages and patients almost always overestimate the impacts on quality of life due to wearing orthodontic appliances [20]. When the clinician understands what patients expect at different steps of treatment, he or she can modify or redesign the communications to reduce patient dissatisfaction finally.

### Summary of evidence

Compared with studies in other disciplines of dentistry, the research of patient expectations from orthodontic treatment is stronger, more systematic and better developed [38]. This is further demonstrated by the different questionnaires, which have been developed and validated to measure the role of expectations in eligible studies. Apart from three survey studies [21, 23, 24], the remaining all adopted standardized questionnaires with a history of validity and reliability tests, even though the standardizations were insufficient in most of studies. In this review, the reliability value was got through test-retest comparison in 2 studies [8, 12]. Internal consistency was got by Cronbach’s alpha calculation in five studies [7, 8, 12, 30, 33]. The measurements of validity were through face validity test in two studies [8, 12] and construct validity test by factor analysis in three studies [7, 8, 33]. All questionnaires except CPQ_11-14_ were designed specifically for orthodontic patients. Although the items in CPQ_11-14_ were applicable to similar domains as the specific questionnaire, the 37 items are still too general and might be a burden for the patients.

Overall, the 14 papers with only one randomized control trial and one cohort study constitute a weak level of evidence on the sub-determiners of expectations. Most of the observational studies with a cross-sectional design described the univariate effect on expectation differences. According to STROBE, studies included in this systematic review are incomplete and inadequate in reporting several items, such as bias description, study size calculation and discussion generalizability, which hampers the evidence level. The most frequent variables were male/female, parents/children, and ethnic differences [22, 25, 26, 29]. Some studies measured the association between expectations and target variables such as gender, age, satisfaction of initial appearance/self-concept/self-image, malocclusion severity, cost/family income and education level [7, 8, 21, 30, 33]. Only two studies utilized multiple factor analysis, which was recommended when the measurement had more than one independent variable [7, 33]. However, without a prospective design and follow up, the hypotheses that patient expectations can affect the treatment related satisfaction, cooperation and compliance cannot be confirmed nor rejected.

### Limitations and future research

The search of literature was restricted to English-language publications, which might introduce citation bias and jeopardize the evidence synthesis. The search process was only limited to electronic databases. Due to the ambiguity in the definitions of expectations and related concepts, selection bias is not unlikely, although effort has been taken to minimize it through the methodology and the utilization of two reviewers. With the heterogeneity in study designs, the results were extracted with an inevitable degree of subjectivity.

The evidence to support the need for orthodontic dentists to clarify patients’ expectations and whether this might assist them in achieving better treatment outcomes and patient satisfactions is weak. Future studies should address this better and consider specific situations the patients may encounter, the kind of expectations they may form and how these expectations would be influenced by sub-determinants such as previous experiences, personal characteristics, social and psychological factors [39]. For example, Bandura’s self-efficacy model is now popular as the theoretical framework in research of patient expectations [40].

To conclude, there is a need for future studies to:

1. Construct the theoretical model of how patients form expectations from orthodontic treatment and demonstrate its determinants and contributing factors both theoretically and experimentally. Stratified or multivariate analysis is recommended.
2. Investigate the nature, extent and clinical implications of the relation between expectations and subsequent treatment outcomes, including evaluations of intervention effectiveness.
3. Investigate the changes or different roles of expectations at different clinical stages, through a longitudinal study design.

## Conclusion

For patients, dental appearance and function improvement are most expected when the orthodontic treatments complete. However, current research is poor in the quality of methodology and results analyses even the standardized questionnaires are adopted. This weakens the evidence of sub-determiners influencing patient expectations. The role of expectations on the orthodontic treatment or relevant treatment factors like patient satisfaction is not investigated at present. Future studies are needed and well-constructed theory is recommended.

## Additional files

Additional file 1:
**Search Strategy.** (PDF 45 kb) Additional file 2: Table S1. Study quality and main findings summary [7, 8, 12, 20–27, 29, 30, 33]. (PDF 121 kb) Additional file 3: Table S2. Parameters of measurement and scale evaluations [7, 8, 12, 20–31, 33]. (PDF 149 kb)
