# Supplementary material for: What are patients’ expectations of orthodontic treatment: a systematic review
Source: BMC Oral Health. 2016 Feb 17;16:19. doi: 10.1186/s12903-016-0182-3 (PMC4756524; doi:10.1186/s12903-016-0182-3)
Supplement: Additional file 1: — Search Strategy. (PDF 45 kb) [file 12903_2016_182_MOESM1_ESM.pdf]

Search Strategy:

#1=expectation;
#2=patient expectation;
#3=expectation satisfaction;
#4=health expectation;
#5=treatment expectation;
#6=expectation outcome;
#7=patient expectations satisfaction;
#8=expectant;
#9=expected value;
#10=expected;
#11=outcome expectations
#12=#1 OR #2 OR #3 OR #4 OR #5 OR #6 OR #7 OR #8 OR #9 OR #10 OR #11
#13=anticipation
#14=#12 OR #13
#15=orthodontic treatment
#16=malocclusion
#17=malocclusion orthodontic
#18=malocclusion treatment
#19=dental malocclusion
#20=#15 OR #16 OR #17 OR #18 OR #19
#21=#20 AND #14
