# Supplementary material for: What are patients’ expectations of orthodontic treatment: a systematic review
Source: BMC Oral Health. 2016 Feb 17;16:19. doi: 10.1186/s12903-016-0182-3 (PMC4756524; doi:10.1186/s12903-016-0182-3)
Supplement: Additional file 2: Table S1. — Study quality and main findings summary [7, 8, 12, 20–27, 29, 30, 33]. (PDF 121 kb) [file 12903_2016_182_MOESM2_ESM.pdf]

Table1. Study quality and main findings summary

A/Y	SROBE	Sample	Design	Method	Study character	Main Finding
Nasr et al. 2011 [27]	Not applicable (N/A)	80<br>(Age: 12-14 years old)<br>First orthodontic consultation appointment	Randomized control trial (RCT)	Scale	1. Expectation Questionnaire [12] test before/after consultation. Comparison between before/after respectively in female and male groups<br>2. Randomization before consultation, then give leaflets.<br>3. Control group (n=40) received information about fluoride; Test group (n=40) had information about orthodontic treatment. Comparison between case/control group	1. There is no significant difference between patient expectations before and after consultation in both groups except two items: the first visit and eating restriction (The after score was higher).<br>2. There were no statistical significant differences in the responses between the genders, except for one item suggested that males believed that they would have to attend more frequently for treatment than females before the intervention<br>3. Information leaflets did not have an immediate impact on patients' expectations of orthodontic treatment
Hiemstra et al. 2009 [26]	12.5	168<br>(Patient age 10-14years)<br>84 new patients and 84 parents	Cross sectional	Scale	1. Questionnaire test [12] before consultation.<br>2. Comparison between children and parents/Dutch samples and UK samples	1. Patients and parents' expectations differ on several aspects<br>2. Boys and girls have only one different expectation of treatments involving jaw surgery<br>3. Dutch patients' expectations are different from UK
Sayers et al. 2006 [12]/ 2007 [25]	13.5	100<br>(50 patients aged 12-14 years old)<br>50 patients and 50 parents	Cross sectional	Scale	Expectation Questionnaire was developed by Sayers in 2006 and have been tested the validity and reliability.<br>1. Questionnaire test [12] before consultation.<br>2. Comparison between children and parents/boys and girls/different Ethnicity	1. Patients and their parents share similar expectations of orthodontic treatment except in dietary and drink restrictions<br>2. Parents are more realistic in their estimation of the duration of treatment and the initial visit.<br>3. Ethnicity is probably one factor leading difference.
Sadek et al. 2015 [22]	12	New patient participants have not received fixed orthodontic appliance treatment and age 12-14 years old; 50 Black British patients and their primary carers and 50 White British patients patients and their primary carers	Cross sectional	Scale	1. Compare expectations of Black British children/their primary cares and White British children/their cares<br>2. Questionnaire was same with above Sayers et al. 2007 [25]	1. Differences in expectations of orthodontic treatment were more common between Black British and White British primary carers, than their children.<br>2. A clinicians understanding of patients and their primary carer's expectations at the start of treatment can help in the quality and delivery of orthodontic care provided.<br>3. White British primary carers had higher expectations at their child' initial appointment and expected dental extractions to be part of the orthodontic treatment plan.
Souza et al. 2013 [24]	12	60 adult students (age 18-25) who had already undergone orthodontic	Cross sectional	Survey	10 questions include patient expectations in relation to the orthodontic treatment and to the orthodontist	Most participants in this study believed that their orthodontic treatment was successfully completed within the predicted time, resulting in esthetical, social, and psychological improvement.

		treatment with fixed appliances or were in the final stages of treatment.				
Rasoo et al. 2012 [23]	12	50 subjects (age 15-30 years), initial motivated	Cross sectional	Survey	9 questions were asked about expectations of the orthodontic treatment and to the orthodontist	Patient's expectations regarding orthodontic treatment were different
Zhang et al. 2007 [20]	18	197 (mean age 13.1years) patients had a perceived need for orthodontic treatment and were about to undergo fixed orthodontic appliance therapy	Cohort	Scale	1. To compare patients' expectations of the impact of wearing fixed orthodontic appliances on life quality with realties experienced over a 6-month period.<br>2. The self-completed Child Perception Questionnaire (CPQ) was modified<br>Expectations were compared with the pretreatment values and reported at 1 week, 1 months, and 6 months after insertion of the fixed appliances	1. This study indicated the impact on quality of life after insertion of fixed orthodontic appliances was consideratelу less than what child patients expected.<br>2. 1 week after the insertion of fixed appliances, Oral symptom (pain mainly) and function limitation compromises were encountered. However, as treatment progressed, oral symptom and function limitation were significantly less compromised than anticipated.<br>EWB and SWB did not compromise as expected, in reality at all-time points of treatment.
**A/Y**	SROBE	**Sample**	**Design**	**Method**	**Study character**	**Main Finding**
Bos et al. 2003 [7]	12	154<br>(Mean age 16 years old)<br>79 males and 74 females<br>First visit	Cross sectional (Questionnaire validation)	Scale	1. Relationship was analyzed to estimate the satisfaction, age, sex on expectations<br>2. After first analysis, subjects were divided into two groups due to the age, group 1: 9-16 years old (n=72), group 2: 17-63 years old (n=28)	1. The dental satisfaction of orthodontic patients is significantly correlated with their expectations about general well-being, improvement of self-image/appearance and future dental health<br>2. These correlations were invariant over gender, but not over age. Age was significantly related to general facial satisfaction and expectations about self-image/appearance.
Wezel et al. 2015 [30]	13	146 subjects who are applying for orthodontic treatment (53 male, 93 female), mean age=19.6 (SD=13.49)	Cross sectional	Scale	1. The aim was to compare the satisfaction and expectations of current patients (mean) with the results of a study 10 years ago [7].<br>2. Factors including gender, age, dentofacial satisfaction were investigated.<br>3. Subjects were separated as the age: 8-16 years old (n=84); 17-60 years old (n=50)	1. Dentofacial satisfaction predicts expectations about orthodontic treatment, especially in the group of subjects aged 17 years and above. For younger patients (<17 years), facial satisfaction was found to be a significant predictor only for expectations about future dental health.<br>2. No correlation was found between gender and expectations or orthodontic treatment.<br>3. In comparsion with the subjects in Bos et al (2003), the subjects in this study had significantly higher expectations about orthodontic treatment.
Tung et al. 1998 [29]	13	75 subjects age 9-12 year old (Mean age 10.85 years). Most were either still in	Cross sectional	Scale	1. Participants are representative of middle- to upper-income groups<br>2. Comparison between the variables of patient and parents' expectations from treatment	1. The mean ratings by children and their parents indicate the greatest expectation of improvement in self-image (e.g., appearance, self-confidence) and oral function (e.g., better chewing and occlusion) but little or no change in the child's

		Phase I therapy (45%) or had completed it (22%).				social life or general health.<br>2. Parents expected greater improvements in the child's self-image, oral function, and social life than did their children.
Petrone et al. 2003 [33]	16	91 parents (patients age ≥18 years, first included in orthodontic treatment, full fees)	Cross sectional	Scale	1. 10-item expectation question tested preceding three months<br>2. Association between treatment fees, subject outcome expectations and malocclusion severity were assessed.	1. The results demonstrated that all expectations were high, except for the ability to brush teeth better after treatment.<br>2. Greater expectations of the benefits of treatment were associated primarily with the level of pretreatment malocclusion. Appearance is less expected.<br>3. Treatment fees were not associated with consumer expectations at all.
Bennet et al. 1997 [8]	18.5	220 parents (Who had sought an initial orthodontic evaluation for children, aged 18 years and younger) and 220 orthodontists	Cross sectional	Scale	1. Two final versions of the questionnaire were created: one for orthodontists and the other for parents.<br>2. Descriptive variables were compared including subjects' income level, educational level, orthodontic experience with orthodontic care, age, and sex.	1. For parents, income, father's education level, and sex of respondent were related to treatment expectations and values.<br>2. Examination of five items (both parents and orthodontists) concerning dental health and self-esteem were not supported by current research findings in orthodontic
Tuncer et al. 2015 [21]	18	491 patients (274 female, 217 male), age 14-22 years, and 399 parents (245 female, 154 male) during consultation or to apply for treatment	Cross sectional	Survey	1. Participants completed a survey (6 items) about preference, needs and expectations about orthodontic treatment and scored their present problems.<br>2. Only one items measuring patients' expectation with three choices (better dental aesthetic; facial esthetic; oral function).<br>3. Education level and angle classifications were evaluated among all parents and only adult patients (over 18)	1. Dental aesthetics was the determinant expectations for orthodontic treatment outcomes for patients (61%) and parents (57.3%).<br>2. Improvement in oral functions was more important for Class III patients than Class I patients (p=0.04).<br>3. Adult patients/parents with higher education (>high school) gave more importance to oral functions as well as dental aesthetics (p=0.031).
