# Supplementary material for: What are patients’ expectations of orthodontic treatment: a systematic review
Source: BMC Oral Health. 2016 Feb 17;16:19. doi: 10.1186/s12903-016-0182-3 (PMC4756524; doi:10.1186/s12903-016-0182-3)
Supplement: Additional file 3: Table S2. — Parameters of measurement and scale evaluations [7, 8, 12, 20–31, 33]. (PDF 149 kb) [file 12903_2016_182_MOESM3_ESM.pdf]

Table2. Parameters of measurement and scale evaluations

Expectation items mainly regarding to treatment per se (8 papers)			
**Author/Year**	**Expectation Parameters**	**Common Factor summary**	**Scale evaluation**
Sayers et al. 2006 [12]/ 2007 [25]<br><br>Hiemstra et al. 2009, [26]<br><br>Nasr et al. 2011 [27]<br><br>Sadek et al. 2015 [22]	1. **At your initial appointment do you expect to**<br>a. Have a brace fitted/b. *Have a check-up and diagnosis*/c. *Have a discussion about treatment* /d. Have x-rays/ e. Have impression/f Have oral hygiene checked<br>2. **What type of orthodontic treatment do you expect?**<br>a. *Braces*, don't know what type? /b. Train track braces? /c. Teeth extracted? /d. Head brace/e. Jaw surgery?<br>**3. Do you think orthodontic treatment will give you any problems?**<br>*Mean range 30-43.7*<br>**4. Do you think wearing a brace will be painful?**<br>*Mean range 43.8-54.1*<br>**5. Do you think orthodontic treatment will produce problems with eating?**<br>*Mean range 47.3-57.5*<br>**6. Do you expect orthodontic treatment to restrict what you can eat or drink?**<br>*Mean range 47.5-67.1*<br>**7. How do you think people will react to you wearing a brace?**<br>*Mean range 48.9-62.9*<br>**8. How long do you expect orthodontic treatment to take**<br>*Don't know / 1-2 years*<br>**9. How often do you think you will need to attend for checking up**<br>*1-2 months*<br>10. **Do you expect orthodontic treatment to**<br>a. *Straighten you teeth*/b. produce a better smile/c. make it easier to eat/d. make it easier to speak/e. Make it easier to keep my teeth clean/f. Improve my chances of a good career? / g. Give you confidence socially	**Initial appointment**<br><br>**Types of treatment**<br><br>**Pain problems**<br><br>**Restriction in function**<br><br>**Restriction in oral hygiene**<br><br>**Social activity**<br><br>**Duration of treatment**<br><br>**Frequency of revisit**<br><br>**Treatment Outcomes**<br><br>**Relationship with Orthodontists**	1. Developed by **Sayers et al., 2006 [12]**<br>2. Semi-structure qualitative interview (30 subjects), a questionnaire was devised form the key themes and concepts identified in the interview.<br>3. Pilot study (10 participants)<br>4. Modified Question, tested again. 22 participants finished test-retest.<br>5. The questionnaire had good face validity<br>6. Internal consistency tested with Cronbach's alpha<br>7. External consistency: Test-retest was statistically significant using Spearman's correlation
Souza et al. 2013 [24]	**Patient expectations in relation to the orthodontic treatment**<br>1. **Orthodontic treatment situation**<br>a. *Finished successful*/b. finished unsuccessfully/c. in treatment, presenting improvement/d. In treatment, with no improvement/e. Not concluded<br>2. **Main reason for searching orthodontic treatment**<br>a. Esthetics/b. *Occlusal disturbances*/c. TMJ pain<br>3. **Treatment duration**<br>a. Quick/b. *As expected*/c. Long due to case complexity/d. Long due to lack of cooperation<br>4. **Treatment results**<br>*a. Very good, esthetic, social and psychological improvement*/b. Satisfactory, but did not change social and psychological life/Regular/c. Bad, did not fulfill expectations/d. Bad, the problem relapsed<br>5. **Type of orthodontic appliance**<br>*a. Metallic*/b. Removable transparent<br>6. **Interference of orthodontic treatment in social relations**<br>a. Partial, compromising smile and relations/b. Partial, without compromising social life/c. *Did not interfere*<br>7. **Interference of orthodontic treatment in oral hygiene**<br>a. No difficulties in oral hygiene/b. Difficulties only in flossing/c. *Difficulties in brushing*		The instrument of data collection was a survey composed of 10 closed-ended questions, drawn from a pilot study conducted at Southwest Bahia State University, in 2004.

	and flossing<br>8. **Main complaint about treatment**<br>a. Duration/b. *pain*/c. oral hygiene/d. esthetics/e. difficulty in chewing/f. difficulty in using orthodontic auxiliaries/high financial cost<br><br>**Patient expectations in relation to the orthodontist.**<br>1. **Patient opinion in relation to the orthodontist**<br>*a. Concerned with patient's health*/b. Concerned with patient's health, but unprepared/c. Concerned with patient's health, but with no skills/d. Little concern with patient<br>2. **Professional which should perform the orthodontic treatment**<br>a. General practitioner/b. *Specialist*	**Proficiency of dentist**<br><br>**Complications**	
Rasool et al. 2012 [23]	**Q1. What are your expectations of first visit?**<br>a. *Examination & diagnosis* b. Discussion c. Get braces d. X-rays e. Impressions f. History taking<br>**Q2. What types of orthodontic treatment do you expect?**<br>a. Extraction of teeth b. *Fixed braces* c. Jaw surgery d. Removable appliances<br>**Q3. What experiences/side effects do you expect from orthodontic treatment?**<br>a. Embarrassment with appliances b. Pain/discomfort in teeth c. Restrict eating/drinking d. Speech problems e. *Difficulty cleaning teeth* f. Problems of gums g. Headache h. Ulcers in mouth<br>**Q4. What benefits of orthodontic treatment do you expect?**<br>a. *Improved appearance of teeth* b. Improved function (chewing, speech) c. Improved smile d. Improved psycho-social well-being e. Break habits f. Gain confidence g. Improved oral and dental health h. Decrease showing too much gums<br>**Q5. What duration of orthodontic treatment do you expect?**<br>a. 4 years b. 3 years c. *2 years* d. 1year e. 6 months f. 2 months<br>**Q6. What reaction do you expect from others/public towards your treatment?**<br>a. None b. *Positive* c. Negative<br>**Q7. What do you expect about frequency of orthodontic visits?**<br>a. 6 months b. 4 months c. 2 months d. *Once a month* e. Twice a month f. Once a week<br>**Q8. What do you expect about the attitude of orthodontist?**<br>a. Supportive b. Neutral c. Critique d. *Confident*<br>**Q9. Which complications of treatment do you expect?**<br>a. Root shortening b. Relapse (return of orthodontic problem) c. Tooth decay<br>d. Pain in jaw muscles and joints e. *Bleeding and swollen gums* f. Mobility of teeth		1. 9 items questionnaire measure was first piloted on 10 subjects who were not part of the study. (No data)<br>2. Modified questionnaire was tested in the 50 subjects and only response number was recorded.
Zhang et al. 2007 [20]	The root of all items in assessment of treatment expectation, patients were asked to rate ''In the next while after you get your braces (during treatment) how often do you expect . . . (i.e, to have bleeding gums)?'	Covering 4 domains:<br>• **Oral symptom**<br>• **Functional limitation**<br>• **Emotional well-being**<br>• **Social well-being**	1. A 37-item Child Perception Questionnaire (CPQ, Jokovic et al., standard, well investigated) [28]<br>2. Each item is scored on a 5-point Likert scale to rate the impact of its oral health status on the particular aspect of Quality of life.

Expectation items mainly regarding to treatment outcomes (6 papers)

Author/Year	Expectation Parameters	Common Factor summary/ Items most Responding	Scale evaluation
Bos et al. 2003 [7]	**Expectation items:**<br>1. General health/ Performance at school or work	Four factors:	1.Items were picked and modified from 1988/1995, Kiyak et al. which is related to Orthoganthic

Tung et al. 1998 [29]<br><br>Wezel et al. 2015 [30]	2. Pain or discomfort in the maxillary joint<br>3. Complaints about maxillary sinus and sinus cavity<br>4. Interacting with people<br>5. Speaking<br>6. Headache<br>7. Sleeping<br>8. Interaction with other<br>9. Breathing<br>10. Feeling in company of other people<br>11. Facial appearance<br>12. Self-confidence<br>13. Appearance<br>14. Self-image<br>15. Dental appearance<br>16. Fit of front teeth<br>17. Fit of molars<br>18. Fit of upper and lower teeth<br>19. Ability to chew food<br>20. Clicking of joints<br>21. Bite off food properly<br>22. Variance explained	**Factor One (10 items): "General well-being**<br>**Factor Two (5 items): "Self-image/appearance"**<br>**Factor Three (4 items): "Future dental health"**<br>**Factor Four (3 items): "Oral function."**	expectation [31]. (The original study have no full text available, however, this questionnaire was cited in lots of papers)<br><br>2. Tung only picked 14 items form the Kiyak et al. 's questionnaire, kept four dimentions [31].<br><br>3. In Bos et al.'s study (22 items):<br><br>● Internal consistency by Cronbach's alpha<br>● Multiple regression analysis was performed to estimate the satisfaction, age, sex on expectations<br>● Principle components analysis of the expectation questionnaire showed 4 factors, accounting for 72% of the total variance.<br><br>4. Wezel et al. (2015) used the same scale with Bos et al. (2003), the Cronbach alpha was presented 0.94 (total scale); 4 subscales were 0.94 (general well-being); 0.91 (self-image); 0.83 (future dental health); 0.85 (oral function).
Petrone et al. 2003 [33]<br><br>Bennett et al. 1997 [8]	**Parents were asked to rate the likelihood of 10 post-orthodontic treatment outcomes:**<br>1. better smile<br>2. more attractive profile,<br>3. better-looking teeth,<br>4. better "bite"<br>5. teeth easier to brush,<br>6. straighter teeth,<br>7. improved self-esteem,<br>8. look better,<br>9. patient pleased with way he or she looks<br>10. straight teeth forever	1. Two factors were found in Petrone's paper:<br>**Factor one: Straightness of the teeth**<br>**Factor two: Appearance**<br><br>2. Four factors in Bennett's<br>**Factor one (10 items): Benefits**<br>**Factor two (6 items): Long term risks**<br>**Factor three (6 items): Short term risks**<br>**Factor four (4 items): Inconvenience**	1. In Petrone's study, items were from the Bennett's items (Factor one, benefits). The overall scale showed high reliability (Cronbach's alpha). Multiple regression model and factor analyses were conducted<br><br>2. Bennett et al.'s study had 52 items. Items for the questionnaire were developed via a qualitative, telephone interview process. Data were analyzed using factor analysis and reliability analysis for scale development, and analysis of variance for preliminary validity assessment. Results of the factor analysis for parents yielded four main factors that together accounted for 45.5% of the variance.
Tuncer et al. 2015 [21]	Only one item was asked:<br>What are your expectations from an orthodontic treatment (More than one answer may be given)<br>a. Better alignment of my/my child's teeth<br>b. Improvement of my/my child's facial esthetics<br>c. Achieve better oral functioning such as chewing, quality of speech, or other (Please specify)	• Dental appearance<br>• Facial esthetics<br>• Oral Function	No data afforded for the validity and reliability of the questionnaire. The author only stated this questionnaire was adapted from a previous study that was found to be valid and reliable after necessary changes were made.

*U mean the main responding
